# Virtual monochromatic versus conventional polychromatic CT images in patients with acute bowel ischaemia

**DOI:** 10.1007/s00330-025-11995-2

**Published:** 2025-09-12

**Authors:** Kilian Rothenbuehler, Ahmad Sayadi, Renaud Troxler, Christine Sempoux, Alban Denys, David Rotzinger, Sabine Schmidt

**Affiliations:** 1 Department of Diagnostic and Interventional Radiology, Lausanne University Hospital (CHUV) and University of Lausanne (UNIL), Lausanne, Switzerland; 2 Department of Medical Imaging, Riviera Chablais Hospital, Rennaz, Switzerland; 3 Department of Pathology, Lausanne University Hospital (CHUV) and University of Lausanne (UNIL), Lausanne, Switzerland

**Keywords:** Abdomen, Acute bowel ischaemia, Dual-energy computed tomography, Virtual non-enhanced phase, Diagnostic value

## Abstract

**Objectives:**

To compare 40 keV virtual monochromatic images (VMIs) with conventionally acquired polychromatic multi-detector CT images (CONV) in patients with acute bowel ischaemia (ABI).

**Materials and methods:**

We retrospectively included 48 consecutive patients (38 males, mean age 69 years) with pathologically proven ABI over 40 months. They underwent portovenous dual-energy multi-detector CT followed by surgery within < 48 h. Nineteen patients had VMIs, and 29 had CONV after non-enhanced acquisition. After dividing the small and large bowel into 10 segments, two radiologists blinded to the exact localisation of the ABI read the VMIs and CONV images separately. After this qualitative analysis, bowel wall density was assessed quantitatively on (virtual) non-enhanced and portovenous images.

**Results:**

Qualitative analysis showed good overall sensitivity (75.9–82%) and specificity (85.9–88.7%) for localising ABI on VMIs and CONV images without significant differences. Inter-observer agreement was important or near perfect (kappa 0.61–0.83). Quantitative analysis revealed significant differences in wall density between healthy and pathological bowel segments for nearly all VMIs and CONV images, not only when analysing the (virtual) non-enhanced and portovenous phases separately, but also when subtracting wall density (portovenous minus (virtual) non-enhanced phase). Wall density delta (healthy minus pathological wall) showed no significant differences between VMIs and CONV images.

**Conclusion:**

VMIs and CONV images had good diagnostic value and important inter-reader agreement for localising ABI using (virtual) non-enhanced and portovenous phases, without significant qualitative or quantitative differences. Therefore, we can rely on the virtual non-enhanced phase instead of additionally acquiring a non-enhanced phase, enabling considerable dose reduction.

**Key Points:**

***Question*** *Can we rely on virtual monochromatic CT images to detect reduced or absent bowel wall enhancement in acute bowel ischaemia?*

***Findings*** *Virtual monochromatic CT images have equal diagnostic value for detecting acute bowel ischaemia as conventionally acquired polychromatic CT images without significant qualitative or quantitative differences.*

***Clinical relevance*** *Significant differences in bowel wall density between healthy and pathological segments on subtraction virtual monochromatic images allow us to rely on the virtual non-enhanced phase instead of acquiring an additional non-enhanced phase in acute bowel ischaemia, leading to dose reduction.*

**Graphical Abstract:**

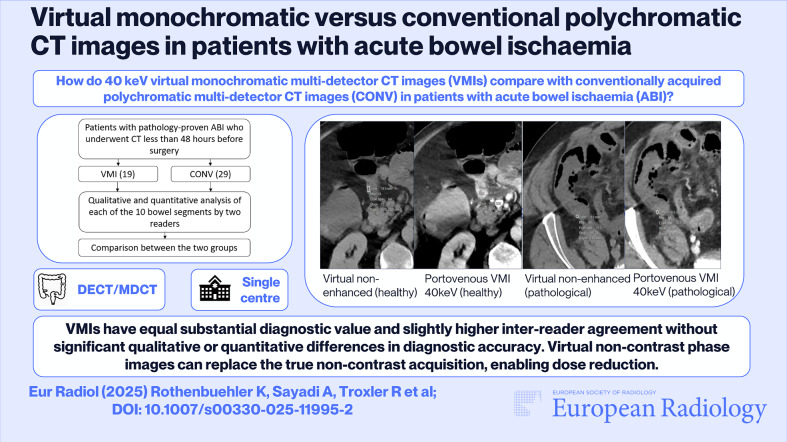

## Introduction

Acute bowel ischaemia (ABI) is an emergency with poor prognosis and a mortality rate of nearly 100%, in the absence of treatment or when treatment is initiated too late [1]. Therefore, prompt diagnosis and the detection of irreversible bowel necrosis are critical [2]. Due to steady availability, fast acquisition time, excellent spatial resolution, and reproducibility of image acquisition, contrast-enhanced multi-detector computed tomography (MDCT) is the imaging modality of choice for ABI, with a reported sensitivity and specificity between 90% and 100% [1, 3–8].

Although several bowel signs have been described as being helpful for detecting ABI on contrast-enhanced MDCT images [1, 8], reduced or absent mural enhancement is known to be a major feature, though variable sensitivity (18–92%) and specificity (50–100%) have been reported [1, 7, 9–11]. The lack of wall enhancement is most easily diagnosed in direct comparison with non-enhanced MDCT images traditionally acquired prior to the contrast-enhanced phase. However, the development of dual-energy computed tomography (DECT) seems to obviate the need for such non-enhanced MDCT acquisition, as virtual non-enhanced images can also be secondarily reconstructed from intravenous contrast medium-enhanced images.

Furthermore, DECT enables the reconstruction of virtual monochromatic images (VMIs) of any energy within a range of 40 keV to approximately 200 keV. Reconstructions at low energy maximise the contrast from the intravenous injection of the iodinated contrast agent as they approach the K-edge energy of iodine, which is 33 keV. Therefore, VMIs at low energy are particularly useful for the distinction between contrast-enhanced and non-enhanced bowel walls, facilitating the diagnosis of ABI.

Multiple authors have reported the general advantages of DECT in abdominal and emergency radiology [12–16], but only a few have focused on the exact benefits of DECT in ABI [17]. To the best of our knowledge, a quantitative analysis of bowel wall enhancement using density measurements on both conventional polychromatic images and VMIs has not yet been performed. Consequently, we designed a retrospective study to qualitatively and quantitatively compare 40-keV VMIs with conventionally acquired polychromatic MDCT images (CONV) in detecting reduced or absent bowel wall enhancement indicative of ABI from any cause. We also aimed to determine whether virtual non-enhanced MDCT images are as reliable as conventional non-enhanced MDCT images in assessing bowel wall density.

## Materials and methods

### Patients

Our study protocol (no 2021-01266) was approved by our institutional ethics committee. Patients’ active consent was waived. This single-centre retrospective study conformed with the Strengthening the Reporting of Observational Studies in Epidemiology (STROBE) guidelines [18]. We consecutively included all adult patients with ABI who were admitted to the emergency department, including intensive care patients between 1 January 2018 and 30 April 2021 and underwent contrast-enhanced abdominal CT during the portovenous phase, followed by surgery within < 48 h. Patients with ABI who only underwent non-enhanced CT or enhanced polychromatic CT acquisition without a non-enhanced phase were excluded, as well as patients < 18 years.

**Fig. 1:**
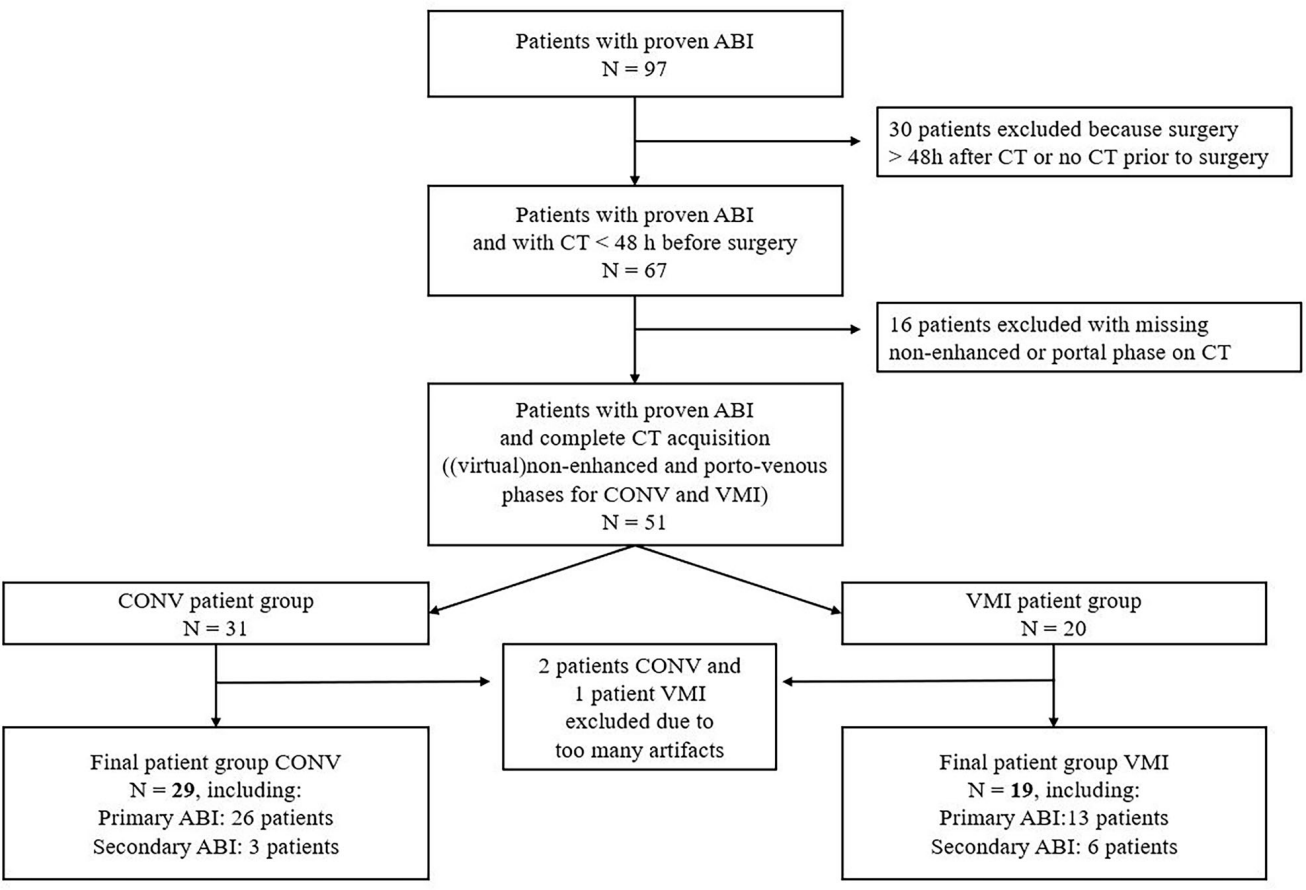
Flow chart of patient inclusion. ABI, acute bowel ischaemia; CONV, conventional polychromatic multi-detector CT images; VMI, virtual monochromatic image

From the initial study population of 97 patients with pathologically proven ABI, 67 patients had undergone IV contrast-enhanced CT acquisition ≤ 48 h prior to surgery. In 51 of these patients, (virtual) non-enhanced acquisition was available in addition to the portovenous phase. After excluding three patients because of inadequate image quality, our final study population consisted of 48 patients. Twenty-nine patients belonged to the CONV group and 19 patients to the VMI group (Fig. 1).

### MDCT

MDCT examinations were performed on a 256-detector row CT system (Revolution, GE Healthcare). The imaging protocol included the whole abdomen and pelvis (diaphragm to pubic symphysis; 120 kV for CONV, fast 80 and 140 kV switching for VMIs; 300–400 mA; table speed, 55 mm per rotation [0.8 s], pitch 1.375, noise index 14). We intravenously injected the iodinated contrast medium Accupaque^®^ (Iohexol, 300 mgI/mL, GE Healthcare, volume in mL = body weight + 30 mL) at a flow rate of 3 mL/s and acquired a portovenous phase (75 s, 1.25/1 mm reconstructed axial slices) in all patients. The portovenous phase consisted of a conventional polychromatic acquisition preceded by a non-enhanced acquisition for the patients of the CONV group. Monochromatic reconstruction at 40 keV of the portovenous phase and a virtual non-enhanced reconstruction were performed for all patients with VMI acquisition. We used the iterative reconstruction algorithm ASiR-V and automatic tube current modulation in all three axes (SmartmA).

The choice to acquire the images in conventional polychromatic mode (CONV) or dual-energy mode (VMI) was based primarily on the clinical judgement of the on-call radiologist at the time, without strict criteria.

Arterial phase images were also acquired in all our patients with suspicion of primary ischaemia, but were not explicitly part of our study design, which focused on evaluating the bowel wall.

### Image analysis

To ensure the reproducibility of our image analysis, the small and large bowel were divided into 10 segments. The small bowel was divided using the umbilicus as a reference point: small bowel loops situated in the left upper quadrant (SB-LUQ), left lower quadrant (SB-LLQ), right upper quadrant (SB-RUQ), and right lower quadrant (SB-RLQ). The large bowel was divided according to the anatomic segments into the caecum, ascending colon, transverse colon, descending colon, sigmoid, and rectum.

### Qualitative analysis

Two radiologists with 15 and 20 years of subspeciality expertise in abdominal imaging, respectively, read CONV (polychromatic non-enhanced and portovenous images) and VMI (virtual unenhanced and 40-keV VMIs in portovenous phase) examinations separately while blinded to the exact localisation of ABI. Diagnostic criteria for ABI were absent or reduced bowel wall enhancement compared with the (virtual) non-enhanced phase. Helpful secondary diagnostic signs were associated bowel dilatation, mesenteric and pericolic stranding, and intestinal pneumatosis ± portovenous gas [19]. These were not analysed further.

### Quantitative analysis

After an interval of 6–8 weeks to reduce recall bias, two radiologists independently measured the bowel wall density quantitatively on (virtual) non-enhanced and portovenous phase VMIs and CONV images in each of the 10 bowel segments using regions of interest (ROIs). ROIs measuring 10 mm², if possible, were placed within the wall of each of the 10 intestinal segments and the mean density, including standard deviation (SD) of each bowel segment recorded in Hounsfield Units (HU) for each patient. The readers were instructed to centre the ROIs on the most pathological bowel segment, to avoid walls with pneumatosis intestinalis, if possible, and to indicate segments with immeasurable walls.

Subsequently, a radiologist reconstructed the portal-venous phase of the VMI examinations in iodine density maps to measure the quantity of iodine uptake in healthy and pathological walls using ROIs.

### Analysis of patient records

One author subsequently reviewed each electronic patient file using our clinical workflow information system Soarian Clinical (Cerner Corporation) and assessed demographic (age, sex) and laboratory parameters (leucocyte count, C-reactive protein (CRP), lactate levels), time between imaging and surgery, and the type of ischaemia. The latter was divided into either primary (i.e., of arterial origin) or secondary (i.e., due to underlying mechanical causes such as strangulation or volvulus resulting from bowel obstruction) ischaemia.

### Statistical analysis

Statistical analyses were performed using R software (version 4.3.1, R Core Team) [20].

Data are presented as numbers and relative percentages. Continuous variables are presented as means ± SD and categorical variables as numbers or proportions. Between-group comparisons were performed using the Wilcoxon rank sum test with continuity correction for continuous variables, and chi-squared with continuity correction or the Fisher test for categorical variables. Statistical differences were considered significant if *p* < 0.05.

For the qualitative analysis of the 10 bowel segments, we calculated the sensitivity, specificity, positive and negative predictive values, and accuracy for each of the two readers using our pathological reference standard, followed by a between-group comparison (CONV versus VMIs).

Inter-reader agreement was assessed by a weighted kappa analysis according to Landis and Koch [21] with the following thresholds: 0–0.2 (very weak agreement), 0.21–0.4 (weak agreement), 0.41–0.6 (moderate agreement), 0.61–0.8 (important agreement), and 0.81–1.00 (near-perfect agreement).

The quantitative analysis comprised three tests:

1. Wall density was compared between healthy and pathological small and large bowel segments, as measured separately by each reader on (virtual) non-enhanced and portovenous VMIs and CONV images.
2. A subtraction analysis was performed to evaluate the difference in wall density between portovenous and (virtual) non-enhanced phases for pathological segments, followed by the same calculation for healthy segments. The results for pathological and healthy segments were then compared separately for VMIs and CONV images, as measured by each reader, and further stratified by small and large bowel.
3. To directly compare mural contrast enhancement conspicuity between VMIs and CONV images, we calculated the delta wall density (defined as the density of the healthy wall in HU minus the density of the pathological wall in HU) as measured by each reader and further stratified by small and large bowel. The delta wall density was furthermore evaluated according to a more standardised approach (defined as (density of the healthy wall in HU minus the density of the pathological wall in HU) divided by the density of the healthy wall in HU). Finally, we calculated the contrast-to-noise ratio (CNR) between the bowel wall (Wall) and mesenteric fat (Fat) on portovenous VMIs and CONV images according to the following formula:
  $${{{\rm{CNR}}}}=\frac{|{{{{\rm{HU}}}}}_{{{{\rm{Fat}}}}}-{{{{\rm{HU}}}}}_{{{{\rm{Wall}}}}}|}{\sqrt{\frac{1}{2}({{{{\rm{SD}}}}}_{{{{\rm{Fat}}}}}^{2}+{{{{\rm{SD}}}}}_{{{{\rm{Fat}}}}}^{2})}}$$

The CNR of the bowel wall was then compared between VMIs and CONV images for both pathological and healthy segments.

## Results

### Patients

Our final study population consisted of 48 patients (38 males, mean age 68.9 ± 15.1 years, range 29–91 years). Twenty-nine patients belonged to the CONV group and 19 patients to the VMI group (Fig. 1). In each patient, ABI was surgically confirmed, after which all except two patients underwent bowel resection. In two patients with extensive ABI, surgery was cancelled due to the fatal outcome. Thus, histopathology was our reference standard for all patients except two, for whom a detailed macroscopic surgical description of each bowel segment was available.

### Demographic, clinical, and laboratory results

There were no significant differences in demographic, clinical, or biological variables between the two patient groups VMI and CONV (Table 1). The lactate level tended to be higher in the CONV group.

**Table 1:** Comparison of demographic, clinical and laboratory variables in patients with acute bowel ischaemia investigated by the two CT techniques

Variables	CONV (*n* = 29)	VMI (*n* = 19)	*p-*value
Age (years)	66.6 ± 15.9	72.4 ± 13.5	0.29
Women	4	6	0.16
Primary/secondary ABI	26/3	13/6	0.12
Delay between imaging and surgery (h)	8.16 ± 9.36	8.68 ± 13.3	0.24
Leucocytes (g/L)	16.63 ± 8.4	19.69 ± 11.2	0.36
C-reactive protein (mg/L)	127.41 ± 115.8	170.38 ± 115.1	0.3
Lactates (mmol/L)	3.83 ± 2.9	2.77 ± 2.4	0.07

Values are given as mean ± standard deviation unless otherwise noted

*CONV* conventionally acquired polychromatic multi-detector CT images, *VMI* virtual monochromatic multi-detector CT images, *ABI* acute bowel ischaemia

In the CONV group (*n* = 29), ABI was of primary origin in 26 patients (89.7%), including 1 patient with concomitant vascular occlusion and 25 patients with non-occlusive mesenteric ischaemia (NOMI). ABI was of secondary origin in 3 patients (10.3%) due to strangulated small bowel obstruction. Surgical resection involved the small bowel in 8 patients, the colon in 16, and both in 5 patients.

In the VMI group (*n* = 19), ABI was of primary origin in 13 patients (68.4%), including 3 patients with concomitant vascular occlusions and 10 with NOMI. ABI was of secondary origin in 6 patients (31.6%), caused by stenosing colon carcinoma (*n* = 2), incarcerated hernia (*n* = 2), or strangulated small bowel obstruction (*n* = 2). Surgical resection involved the small bowel in 8 patients, the colon in 6 patients, and both in 5 patients.

### Qualitative analysis

The two radiologists performed a qualitative analysis of a total of 480 bowel segments (290 in CONV and 190 in VMI acquisition). Table 2 shows sensitivity, specificity, positive predictive value, negative predictive value, and accuracy for each technique, each bowel segment, and each reader for CONV versus VMIs. The overall sensitivity and specificity for the two readers ranged from 75.9% to 82% and from 85.9% to 88.7%, respectively, without any significant differences between the two techniques (*p* = 0.55 and *p* = 0.5, respectively).

**Table 2:** Qualitative analysis of 480 bowel segments for the detection of ABI

	CONV					VMI				
	Sens	Spec	PPV	NPV	Acc	Sens	Spec	PPV	NPV	Acc
Reader 1										
SB-LUQ	100	95.83	83.29	100	96.55	80	85.71	66.65	92.31	84.21
SB-LLQ	77.78	90	77.75	90.01	86.21	90.91	100	100	99.89	94.74
SB-RUQ	80.00	95.83	79.95	95.84	93.11	100	71.43	55.54	100	78.94
SB-RLQ	83.33	82.35	76.94	87.49	82.76	91.67	85.71	91.68	85.69	89.48
Caecum	73.33	71.43	73.31	71.45	72.41	83.33	84.62	71.45	91.66	84.21
Asc colon	66.67	58.82	53.35	71.41	62.07	62.50	81.82	71.42	75.00	73.69
Trans colon	71.43	72.73	45.40	88.91	72.41	80.00	78.57	57.12	91.67	78.95
Desc colon	100	100	100	100	100	75.00	80.00	50.37	92.20	78.94
Sigmoid	66.67	95.00	85.69	86.38	86.22	66.67	81.25	40.02	92.85	78.95
Rectum	50.00	92.00	50.01	92.00	86.20	50.00	94.12	49.93	94.13	89.49
All segments	77.01	87.32	72.25	89.86	84.23	81.97	85.91	70.37	92.10	84.76
Reader 2										
SB-LUQ	40.00	100	100	88.92	89.68	100	85.71	71.41	100	89.47
SB-LLQ	77.78	95.00	87.48	90.49	89.66	90.91	100	100	88.89	94.74
SB-RUQ	80.00	100	100	96.01	96.56	100	92.86	83.32	100	94.74
SB-RLQ	83.33	82.35	76.94	87.49	82.76	91.67	100	100	87.48	94.73
Caecum	73.33	92.86	91.66	76.49	82.76	66.67	76.92	57.17	83.32	73.68
Asc colon	75.00	76.47	69.25	81.24	75.86	75.00	81.82	74.22	82.43	79.02
Trans colon	71.43	59.09	35.67	86.69	62.06	80.00	57.14	39.98	88.90	63.15
Desc colon	100	95.00	89.99	100	96.55	50.00	73.33	33.40	84.58	68.41
Sigmoid	66.67	95.00	85.69	86.38	86.22	33.33	100	100	88.88	89.47
Rectum	75.00	88.00	50.01	95.65	86.21	50.00	100	100	94.46	94.75
All segments	77.86	88.67	74.16	89.55	84.83	80.33	86.05	73.13	90.25	84.21

*ABI* acute bowel ischaemia, *CONV* conventionally acquired, polychromatic multi-detector CT images, *VMI* virtual monochromatic images, *Sens* sensitivity, *Spec* specificity, *PPV* positive predictive value, *NPV* negative predictive value, *Acc* accuracy. *SB* small bowel, *LUQ* left upper quadrant, *LLQ* left lower quadrant, *RUQ* right upper quadrant, *RLQ* right lower quadrant, *Asc* ascending, *Trans* transverse, *Desc* descending

The comparison between the small and large bowel for ABI localisation was generally non-significant, with three exceptions: First, reader 2 assessed ABI in the small bowel with higher sensitivity than ABI in the large bowel on VMIs (*p* = 0.008). In addition, both readers assessed ABI in the small bowel with a higher specificity than ABI in the large bowel on CONV images (*p* = 0.01 and *p* = 0.001, respectively).

The overall interobserver agreement between the two readers was important (kappa 0.73). For the analysis of CONV images, the agreement was important (kappa 0.72), and for VMIs, it was nearly perfect (kappa 0.83).

Qualitative image analysis also revealed pneumatosis intestinalis in 7 and 10 patients and free abdominal fluid in 22 and 19 patients of the CONV and VMI group, respectively.

### Quantitative analysis

From the 480 bowel wall segments (290 in CONV and 190 in VMI mode), reader 1 considered 66 wall segments (13.7%, 22 CONV, 44 VMI) and reader 2 considered 45 wall segments (9.4%, 28 CONV, 17 VMI) as non-assessable, mostly because the walls were too thin, due to extreme luminal dilatation.

**Fig. 2:**
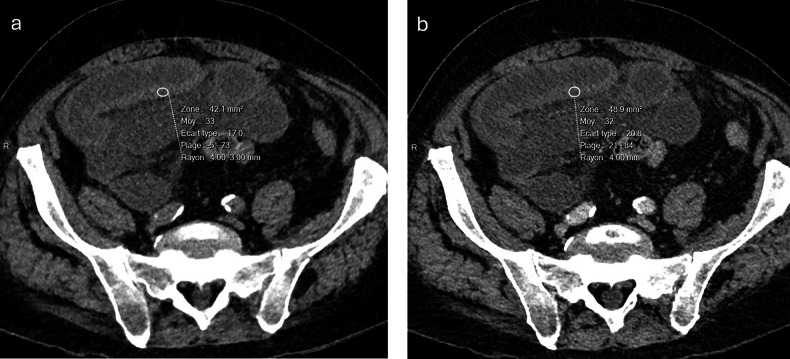
Axial, conventionally acquired, polychromatic non-enhanced (**a**) and portovenous (**b**) multi-detector CT images demonstrate acute bowel ischaemia resulting from acute small bowel obstruction in an 88-year-old male patient. Quantitative analysis of the bowel wall revealed no enhancement during the portovenous phase (**b**), with a mean density of 33 Hounsfield units (HU) in **a** and 32 HU in **b**

**Fig. 3:**
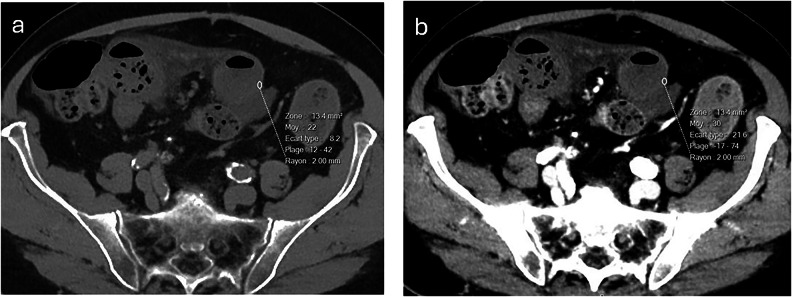
Axial 40 keV virtual monochromatic non-enhanced (**a**) and portovenous (**b**) multi-detector CT images demonstrate acute bowel ischaemia resulting from acute small bowel obstruction in an 86-year-old male patient. Quantitative analysis of the bowel wall revealed only minimal change in the mean bowel wall density during the portovenous phase (**b**), with a mean density of 30 Hounsfield units (HU) in **b** compared to 22 HU in **a**

Significant differences (*p* < 0.05) in wall density between healthy and pathological bowel segments were detected on non-enhanced and portovenous acquisitions for both CONV (Fig. 2) and VMI examinations (Fig. 3), except for reader 2 when comparing the non-enhanced healthy small bowel segments with the pathological small bowel segments on VMIs (Table 3). ROI subtraction analysis yielded significant differences in wall density throughout all bowel segments for both readers and for each acquisition mode (Table 3).

**Table 3:** Quantitative analysis of bowel wall density

	Reader 1		Reader 2	
	*p*-values			
Wall densities	CONV	VMI	CONV	VMI
Small bowel				
(Virtual) non-enhanced phase: healthy vs pathological wall	**< 0.01**	**< 0.01**	**< 0.01**	0.119
Portovenous phase: healthy vs pathological wall	**< 0.01**	**0.011**	**< 0.01**	**0.033**
Large bowel				
(Virtual) non-enhanced phase: healthy vs pathological wall	**< 0.01**	**< 0.01**	**0.03**	**0.042**
Portovenous phase: healthy vs pathological wall	**< 0.01**	**< 0.01**	**< 0.01**	**< 0.01**
Subtraction analysis				
Small bowel				
Portovenous − (virtual) non-enhanced pathological wall vs portovenous − (virtual) non-enhanced healthy wall	**< 0.01**	**< 0.01**	**< 0.01**	**< 0.01**
Mean density (HU): healthy − pathological wall	7.58	14.55	7.64	10.64
Large bowel				
Portovenous − (virtual) non-enhanced pathological wall vs portovenous − (virtual) non-enhanced healthy wall	**< 0.01**	**< 0.01**	**< 0.01**	**< 0.01**
Mean density (HU): healthy − pathological wall	5.27	25.26	4.43	23.72

Significant *p*-values are in bold

*HU* Hounsfield units, *CONV* conventionally acquired polychromatic multi-detector CT images, *VMI* virtual monochromatic images

We found no significant differences when comparing the delta wall density (defined as the density of the healthy wall minus the density of the pathological wall expressed in HU) between the CONV and VMIs, stratified by small and large bowel (Table 4). Notably, for reader 1, a trend favouring VMIs was observed because of higher delta values for the small and large bowel than those measured on CONV images. The standardised approach (defined as (density of the healthy wall in HU minus the density of the pathological wall in HU) divided by the density of the healthy wall in HU) showed a significant difference in favour of CONV for the small bowel evaluation of reader 1 and a significant difference in favour of VMI for the large bowel evaluation of reader 2. The CNR, computed on the basis of the HU for the bowel wall and adjacent mesenteric fat, did not reveal any significant differences between CONV and VMIs for healthy and pathological small bowel segments. The CNR of healthy and pathological large bowel segments, as measured by reader 1, was significantly in favour of VMIs (Table 5).

**Table 4:** Comparison between wall densities measured on CONV and VMIs by means of delta (healthy – pathological) values

	Reader 1		Reader 2	
	HU	*p*-values	HU	*p*-values
Small bowel wall				
CONV (healthy − pathological) vs VMI (healthy − pathological)	24 ± 42.5 vs 51 ± 117.5	0.093	33.5 ± 47.0 vs 25.0 ± 78.0	0.611
Large bowel wall				
CONV (healthy − pathological) vs VMI (healthy − pathological)	17.0 ± 41.0 vs 20.5 ± 122.5	0.082	9.0 ± 44.0 vs 10 ± 116.5	0.423

Normalised	Reader 1		Reader 2	
		*p*-values		*p*-values
Small bowel wall				
CONV (healthy − pathological/healthy) vs VMI (healthy − pathological/healthy)	0.4 ± 0.4 vs 0.2 ± 0.5	**0.019**	0.4 ± 0.3 vs 0.3 ± 0.4	0.533
Colon wall				
CONV (healthy − pathological/healthy) vs VMI (healthy − pathological/healthy)	0.03 ± 0.6 vs 0.21 ± 1.1	0.459	0.05 ± 1.0 vs 0.4 ± 1.2	**0.009**

Significant *p*-values are in bold

*CONV* conventionally acquired polychromatic multi-detector CT images, *VMI* virtual monochromatic images, *HU* Hounsfield Units

**Table 5:** Contrast-to-noise ratio of healthy and pathological bowel walls (related to mesenteric fat)

	Reader 1		Reader 2	
Small bowel		*p*-values		*p*-values
Healthy CONV vs healthy VMI	11.0 ± 2.9vs 11.8 ± 4.3	0.21	10.8 ± 2.8 vs 10.5 ± 4.0	0.91
Pathological CONV vs pathological VMI	8.7 ± 2.8 vs 10.6 ± 2.8	0.12	8.8 ± 4.5 vs 9 ± 3.2	0.32
Large bowel				
Healthy CONV vs healthy VMI	6.4 ± 5.5 vs 8.9 ± 4	**< 0.01**	9.1 ± 3.4 vs 10.2 ± 3.6	0.10
Pathological CONV vs pathological VMI	8.6 ± 2.3 vs 10.8 ± 2.9	**< 0.01**	9.1 ± 2.3 vs 9.8 ± 3.8	0.07

Significant *p*-values are in bold

*CONV* conventionally acquired polychromatic multi-detector CT images, *VMI* virtual monochromatic images

Finally, iodine quantification in the bowel wall assessed on the iodine density maps of the VMI acquisitions (*n* = 19) yielded significant differences between the healthy and pathological segments for both the small bowel (healthy wall = 2.39 ± 0.58 mg/mL, pathological wall = 1.1 ± 0.42 mg/mL, *p* < 0.001) and the large bowel (healthy wall = 2.51 ± 0.65 mg/mL, pathological wall = 1.34 ± 0.6 mg/mL, *p* < 0.001), because of decreased iodine content in the ischaemic walls.

## Discussion

In this retrospective study, we showed that the diagnostic value of 40-keV VMIs for detecting ABI was equal to that of CONV images, with good sensitivity and specificity among all readers. Quantitative analysis revealed significant differences in wall density between healthy and ischaemic bowel segments, which were nearly consistently detected on both (virtual) non-enhanced, portovenous acquisitions and subtracted images. Therefore, we conclude that virtual non-enhanced images are as reliable as (polychromatic) non-enhanced images when compared to portovenous images for detecting and localising reduced or absent wall contrast enhancement.

Two of our results demonstrated a slight superiority of VMIs over polychromatic acquisition. We observed a trend favouring VMIs, as the measurements of reader 1 yielded higher delta values for the small and large bowel, and we found a significantly higher CNR for healthy and pathological large bowel segments when assessed by reader 1.

Several previous authors addressed this topic, concluding that DECT significantly improves the conspicuity of the ischaemic bowel compared with conventional CT by increasing attenuation differences between ischaemic and perfused segments on low-energy images [17, 22–25]. However, only Lourenco et al directly compared conventional polychromatic images with virtual monoenergetic (40 keV) images [22]. Retrospectively including 60 patients with suspected ABI, these authors reported improved diagnostic accuracy, better conspicuity and a higher interobserver agreement for 40-keV VMIs than with conventional polychromatic acquisition. We also observed a slightly higher interobserver agreement for VMIs (0.83 vs 0.72). As in Lourenco et al, we chose to reconstruct our VMIs at 40 keV, providing maximal mucosal enhancement and facilitating the detection of attenuation differences. The qualitative analysis of our readers did not reveal significant diagnostic differences between CONV and VMI, which partly aligns with Lourenco et al’s results [22]. The slightly lower diagnostic value of our qualitative analysis compared to that described by Lourenco et al may be attributed to the greater challenge of precisely localising ABI in a patient as opposed to determining whether the patient has ABI without the need for exact localisation.

Despite quantifying the iodine wall concentration and observing significant differences between healthy and pathological bowel segments, we did not include iodine density maps in our image analysis. Xu et al found no significant difference in ABI diagnosis or diagnostic confidence and even decreased sensitivity and negative predictive value using supplementary iodine map images added to the analysis of VMIs. Therefore, they advised against the systematic use of iodine density maps in clinical routine [24]. According to Wu et al, false linear iodine densities may appear along gas/water or gas/soft-tissue interfaces on these maps, which can simulate mural iodine enhancement [26].

Our study has several limitations. First, though we aimed at having a pathological reference standard for all patients, two of them did not undergo bowel resection, requiring us to rely on the detailed surgical report. Second, our study was retrospective and conducted at a single centre. Third, we included patients with primary and secondary acute bowel ischaemia, which introduces heterogeneity in disease aetiology and may affect the interpretation of imaging findings. Nonetheless, this study design may better reflect the true clinical scenario in which patients are encountered. Fourth, the relatively small number of patients in the VMI group may compromise the robustness of the statistical analysis. However, the approach of a segment-by-segment analysis enabled us to collect more data, substantially improving the statistical results.

Fourth, we did not include the arterial phase in our image analysis, although it was acquired in all our patients with suspected primary ABI. Indeed, the arterial phase is essential for vessel changes occurring in ABI; however, our image analysis focused on bowel wall assessment, for which the portovenous phase needs to be considered [1]. Finally, bowel wall enhancement was the sole parameter used to localise ABI, rather than incorporating other, more indirect features, such as bowel wall thickness, pneumatosis intestinalis, portal-venous gas or vascular signs. Indeed, in a recent meta-analysis, Reimtal Blaser et al pointed out that none of the non-vascular features alone was sufficiently reliable to diagnose ABI or its progression to transmural necrosis, whereas a combination of different radiological features conveyed a potential [27]. However, in many cases of ABI, the vascular occlusion is not detected or present, highlighting the pathophysiological variety of AMI [27]. This also applies to our study population, including 4 patients (8.3%) with vascular occlusion only.

In conclusion, our comparison of VMIs with CONV images for localising ABI revealed that VMIs have substantial diagnostic value and slightly higher inter-reader agreement without significant qualitative or quantitative differences in diagnostic accuracy. In addition, we found significant differences in bowel wall density between healthy and pathological segments on subtraction images in our two groups. Thus, we can rely on the virtual non-contrast phase instead of acquiring an additional non-contrast phase when ABI is suspected, which will enable considerable dose reduction in the future.

## Abbreviations

ABI: Acute bowel ischaemia
CNR: Contrast-to-noise ratio
CONV: Conventionally acquired polychromatic multi-detector CT images
CT: Computed tomography
DECT: Dual-energy computed tomography
HU: Hounsfield unit
MDCT: Multi-detector computed tomography
NOMI: Non-occlusive mesenteric ischaemia
ROI: Region of interest
SB-LUQ: Small bowel left upper quadrant
SB-LLQ: Small bowel left lower quadrant
SB-RLQ: Small bowel right lower quadrant
SB-RUQ: Small bowel right upper quadrant
SD: Standard deviation
VMI: Virtual monochromatic image
